# Fractured Neck of Femur Clinical Pathway Use in Tasmanian Emergency Departments: A Retrospective Study

**DOI:** 10.1111/1742-6723.70155

**Published:** 2025-10-14

**Authors:** Innocent Tawanda Mudzingwa, Sarah Jane Prior, Phoebe Griffin, Emma Tavender, Viet Tran

**Affiliations:** ^1^ Tasmanian School of Medicine University of Tasmania Hobart Tasmania Australia; ^2^ Tasmanian School of Medicine University of Tasmania Burnie Tasmania Australia; ^3^ Tasmanian School of Medicine University of Tasmania Launceston Tasmania Australia; ^4^ Emergency Research, Murdoch Children's Research Institute; Departments of Paediatrics and Critical Care University of Melbourne; Australian Catholic University Melbourne Victoria Australia

**Keywords:** emergency department, fractured NOF pathway, patient and organisational outcomes, regional ED

## Abstract

**Introduction:**

The emergency department (ED) has a critical role in initiating early treatment for fractured neck of femur (fractured NOF). Delayed treatment is associated with adverse outcomes. The primary objective of this study was to retrospectively examine the use of a fractured NOF formal pathway document. Secondary outcomes assessed pathway compliance, impact on waiting times, and differences across Tasmanian EDs to guide local quality improvement.

**Study Design:**

Descriptive retrospective study with random sampling of 200 fractured NOF cases across three regional hospital EDs.

**Results:**

Of the 195 fractured neck of femur (NOF) ED presentations across three sites, 155 (79.5%) used the pathway. Nerve block administration showed a statistically significant association with pathway use, with a relative increase of +44.9% (95% CI: +15.0% to +83.0%, *p* < 0.000001). MiniCog screening showed a notable relative increase of +93.5% (95% CI: +1.0% to +271.0%) but did not meet the significance threshold. MiniCog assessment differed significantly between hospitals H1 versus H2 and H1 versus H3 (*p* < 0.00001). Goals of care completion showed some variation (*p* = 0.0033) but was not statistically significant. Pathway use did not significantly affect waiting times, length of stay, or representation rates. Hospital comparisons revealed significant differences in waiting times and length of stay.

**Conclusions:**

This study suggests that pathway use was associated with significant increases in the administration of nerve blocks. Significant differences in pathway use, element completion, and associated waiting times were reported between hospitals. Findings support the pathway's role in standardising care and guiding targeted quality improvement efforts.

## 1 Introduction

A fractured neck of femur (NOF) is a critical injury, especially in older adults. It involves a break in the proximal femur within the confines of the hip joint capsule, specifically in the region just inferior to the femoral head. In most cases, it is life‐changing and can be life‐threatening. Fractured NOF injuries are expected to increase from 1.7 million in 1990 to 6.3 million by 2050 because of rising life expectancy worldwide [1]. Prolonged patient triage and evaluation, diagnosis, and treatment, as well as delays in the administration of necessary medications such as analgesics, contribute to poorer patient outcomes, leading to substantial morbidity and mortality [2, 3, 4]. The ED has a critical role in initiating early treatment for fractured NOF and enabling prompt transfer to orthopaedic wards [5]. Clinical pathways have been shown to be an effective approach for enhancing operational efficiency and quality of care [6].

Hip fractures are one of the top three causes of emergency admissions involving surgery in Australia [7]. They are also among the most widely implemented clinical pathways in emergency care settings globally [8]. Up to a third of elderly patients who visit the ED with fractured NOF have cognitive impairment and present with pain‐induced delirium [9, 10]. There is strong evidence showing that peripheral nerve blocks for hip fractures are safe and effective in ED settings in providing immediate site‐specific analgesia, reducing the need for opioids, and potentially decreasing delirium risk in elderly patients [11]. Pain management for patients presenting with fractured NOF is therefore important to prevent complications like delirium, depression, and delayed recovery. However, pain management practices have been reported to vary across jurisdictions and hospitals in Australia [1, 12]. The risk of death for people with fractured NOF within 12 months following an accident is 3.5 times higher than for those without [13]. The Australia and New Zealand hip fracture registry (ANZHFR) provides guidance and recommendations on hip fracture treatment. Our study focused on some of its recommendations in the ED setting. Healthcare research is increasingly focusing on reducing variability in care, thereby improving patient outcomes [14]. Clinical pathways support this by standardising care and aligning decisions with evidence‐based practices [6]. They enhance quality by structuring interventions and decision points [15], reducing risk and duplication. Reported potential benefits include shorter treatment times, ED stays, and reduced differences in care [8].

Although the use of clinical pathways in the healthcare sector has increased broadly, it is still unclear why they are not used more often in complex settings like the ED [16]. Therefore, it is important to understand how the fractured NOF pathway is being used in the ED setting and, in this instance, regional EDs, where there has been little research to date. A regional ED in Australia is located outside major metropolitan areas, serving rural or regional communities. The primary objective of this study was to retrospectively examine the use of the fractured NOF pathway, focusing on whether the formal pathway document was being used or not. Secondary outcomes assessed pathway compliance, impact on waiting times, and differences across Tasmanian EDs to guide local quality improvement.

## 2 Materials and Methods

### 2.1 Study Design

This descriptive retrospective study analysed previously collected data to examine characteristics, outcomes, and patterns within the clinical setting, using a random sample of 200 fractured neck of femur (NOF) cases. The sample was selected from eligible ED presentations based on diagnosis of fractured NOF on patients discharged from ED for the period 01 January 2021 to 31st December 2021. Data were extracted from digital medical records.

### 2.2 Setting and Population

Tasmania is considered regional under the Australian Standard Geographic Classification Remoteness Areas. The sample included presentations of all ages who presented with a diagnosis of fractured NOF at one of the three hospitals in the Tasmanian Health Service (THS), each in a different region. The statewide fractured NOF pathway was introduced to improve the quality and safety of acute care in Tasmania. Implemented in July 2019, it is a paper‐based protocol used across all Tasmanian EDs to address local clinical challenges. It outlines the appropriate order of clinical interventions, deadlines, and decision points for this patient cohort.

### 2.3 Data Collection

Data were collected from three hospitals in the Tasmanian Health Service, with approximately 100 presentations from hospital H1 and 50 each from hospitals H2 and H3 included in the study. The study used routinely collected all admissions data (425 presentations for the period under review) to assess the representativeness of our sample based on ED diagnostic codes across three hospitals. This ensured that the sample accurately reflected the characteristics of the population from which it was drawn.

This study utilised data from the IMplementing Pathways for Acute Care in Tasmania (IMPACT) initiative, which used standardised data collection procedures across all Tasmanian EDs using the web‐based tool, REDCap, hosted at the University of Tasmania. REDCap supports research data capture with a user‐friendly interface, audit trails, automated data exports to statistical tools, and integration with external sources, ensuring efficient and validated data management throughout the research process [17]. All fractured NOF presentations for 2021 were assigned a random number in Excel using the RAND() function; the records were then sorted from smallest to largest, and the first 100, 50, and 50 records for H1/H2/H3 were selected for data collection, respectively. There were two independent people (research assistants) extracting data from all three sites with oversight and regular meetings to discuss data collection. Research assistants collected data using explicit definitions for the pathway variables from a published IMPACT data dictionary onto an Excel spreadsheet. Researchers extracted clinical indicators and outcome elements from de‐identified eligible digital patient medical records. These were identified through triage descriptors and confirmed via chart reviews. Key variables included are summarised in Table 1 and included ED length of stay (ED LOS), 28‐day ED re‐presentations, and hospital length of stay (Hosp LOS). Completion of key clinical milestones (elements) defined by the pathway was captured for each presentation. Elements included x‐ray, blood investigations, goals of care, cognitive assessment, pain management, and nerve blocks, and whether data indicated documentation or evidence of referral to orthopaedics. Pain management included the record of, or charting of, oral paracetamol and/or IV/subcutaneous morphine or fentanyl. If a blank medication chart was scanned into the digital record, it was interpreted that the patient did not receive any medications. Absence of documentation or evidence of referral to orthopaedics was considered as not completed. Therefore, in our study, no element completion was interpreted as either not completed or absence of evidence in the digital record. A pathway was considered used when the formal pathway document was either partially or entirely completed. Partial use of the pathway included documentation of patient characteristics, waiting times, and some element completion. Full use referred to full utilisation of the document with all sections completed. A scanned blank pathway document was considered non‐use. Missing data were supplemented through additional chart reviews, and any de‐identified records with unresolved key missing data were excluded. Researchers resolved discrepancies in data transcription collaboratively to ensure accuracy.

**TABLE 1:** Patient, organisational outcomes, and fractured neck of femur (fractured NOF) clinical pathway elements.

Patient outcomes	Organisational outcomes	Fractured NOF clinical pathway elements
Waiting times Pain management 28‐day representation	ED LOS Hosp LOS	x‐ray Blood investigations Goals of Care (GoC) Mini cognitive test (MiniCog) Pain management – Paracetamol and other Nerve block Documentation of referral to orthopaedics.

## 3 Data Analysis

Data were analysed in R Studio (RStudio 2024.09.1 + 394). Continuous variables (age, waiting times, and duration of stay) were summarised by the mean, whilst categorical variables were summarised by the frequency and percentage.

### 3.1 C*ategorical Data*

Chi‐square was used for interventions with sufficient counts and variability, whilst Fisher's exact test was used for interventions with low counts and skewed distribution. We reduced the probability of making a Type I error using the Bonferroni correction to our 0.05 significance level based on 19 statistical tests in the study (0.05/19 = 0.0026). Any *p*‐value less than 0.0026 was considered statistically significant. Risk difference quantified the absolute change in outcome rates between pathway and non‐pathway groups, while relative difference expressed the proportional change, indicating how much more or less common an outcome was in the pathway group.

### 3.2 Continuous Data

Welch's t‐test was used to compare means between two independent groups. It is robust to unequal variances and sample sizes, making it suitable for this dataset. The observations were obtained independently and randomly from the population defined by the factor levels. Any *p*‐value less than 0.0026 was considered statistically significant after Bonferroni correction.

## 4 Results

Table 2A provides a summary of how our sample compared to all admissions data for fractured NOF in the same period represent.

**TABLE 2A:** Representativeness of sample.

	All admissions data (2021)	Sample data (2021)
Presentations	425	195
Mean age	78.9	78.8
	**N (%) = proportion of the total presentations**	**N (%) = proportion of the total study sample presentations**
Pathway use	346 (81.4%)	155 (79.5%)
**Age group**		
59 and under	*29 (7%)*	12 (6%)
60–69	*52 (12%)*	22 (11%)
70–79	*103 (24%)*	46 (23%)
80–89	*153 (36%)*	64 (32%)
90+	*88 (21%)*	45 (23%)
**Gender**		
Female (%)	266 (63%)	122 (62%)
**Hospital**		
*H1*	*196 (47%)*	100 (51%)
H2	*137 (33%)*	50 (25%)
H3	*86 (21%)*	47 (24%)
**Arrival time**		
0001–0800	*62 (15%)*	30 (15%)
0801–1600	*181 (43%)*	82 (42%)
1601–0000	*176 (42%)*	85 (43%)
**Triage category**		
Cat 2	*39 (9%)*	20 (10%)
Cat 3	*309 (73%)*	132 (67%)
Cat 4	*73 (17%)*	43 (22%)

### 4.1 Participant Characteristics

A total of five presentations were excluded from the final analysis due to missing data or duplication. Three presentations were excluded from the final analysis as these patients were transferred immediately from one hospital ED to another hospital ED on presentation. One presentation was a transfer that had missing data, and another had x‐rays and bloods completed in a private hospital before being transferred to the public ED. This left 195 presentations for analysis in this study.

Table 2B provides a summary of the overall baseline participant characteristics for fractured NOF presentations. The mean age of the participants was 79.4 years, ranging from 18 to 104 years. The predominant age range was 80–89 years, with 72 (37%) of the presentations. Most of the presentations were female (62%). More presentations (44%) arrived between 1600 and midnight hours, which was marginally higher than those who arrived during the day (42%). Category three was the predominant triage category, with 67% of all presentations in the sample.

**TABLE 2B:** Overall baseline participant characteristics for #NOF presentations.

	Overall study sample	Pathway used	Pathway not used
**Presentations**	195	155	40
**Mean age (years)**	79.4	79.3	79.4
	**N (%) = proportion of the total study sample presentations**	**N (%) = proportion of the total presentations that used the pathway**	**N (%) = proportion of total presentations that did not use pathway**
**Age group**			
59 and under	12 (6%)	8 (5%)	4 (10%)
60–69	22 (11%)	16 (10%)	6 (15%)
70–79	46 (24%)	36 (23%)	10 (25%)
80–89	72 (37%)	51 (33%)	13 (33%)
90+	43 (22%)	36 (23%)	7 (18%)
**Gender**			
Female (%)	120 (62%)	94 (61%)	26 (65%)
**Hospital**			
H1	98 (50%)	78 (50%)	20 (50%)
H2	50 (26%)	39 (25%)	11 (28%)
H3	47 (24%)	38 (25%)	9 (22%)
**Arrival time**			
0001–0800	29 (15%)	26 (17%)	4 (10%)
0801–1600	81 (42%)	64 (41%)	16 (40%)
1601–0000	85 (44%)	65 (42%)	20 (50%)
Triage category *(only summarised the three most common categories)			
Cat 2	20 (10%)	14 (9%)	6 (15%)
Cat 3	131 (67%)	109 (70%)	22 (55%)
Cat 4	35 (18%)	31 (20%)	11 (28%)

### 4.2 Use of Formal Pathway Document

The total number in our sample that utilised the pathway across the three hospitals was 155 (79.5%), comprising 107 (69%) partial use and 48 (31%) use of the pathway document in its entirety. **H3** had the highest use of the pathway document in its entirety, accounting for 87% of presentations at this hospital.

### 4.3 Completion of Pathway Elements

A comparison of pathway element completion between patients for whom the pathway was used (*n* = 155) and those for whom it was not used (*n* = 40) was conducted. Table 3 summarises the overall completion rate of elements of the pathway when a pathway document was used and when it was not across all three hospitals. Nerve block administration showed a statistically significant association with pathway use, with a relative increase of +44.9% (95% CI: +15.0% to +83.0%, *p* < 0.000001). MiniCog screening showed a notable relative increase of +93.5% (95% CI: +1.0% to +271.0%) but did not meet the significance threshold. These results suggest the pathway may improve the use of specific clinical practices, particularly in nerve blocks and cognitive screening. Most elements showed only small differences between groups where the pathway was used versus not used. These differences were not statistically significant, as shown by the confidence intervals for absolute differences and relative differences.

**TABLE 3:** Element completion when pathway was used and when it was not.

	Pathway used (*n* = 155)	Pathway not used (*n* = 40)	Absolute Difference % (95% CI)	Relative Difference % (95% CI)	*p*‐value
Overall element completion	137 (88%)	32 (80%)	+8.4% (−5.0% to +21.8%)	+10.5% (−6.0% to +30.0%)	0.258
X‐ray investigations	155 (100%)	40 (100%)	0.0% (0.0% to 0.0%)	0.0% (0.0% to +0.0%)	1.000
Blood investigations	154 (99%)	39 (98%)	+1.9% (−3.1% to +6.9%)	+1.9% (−3.0% to +7.0%)	0.369
Goals of care (GoC)	145 (94%)	38 (95%)	−1.5% (−9.2% to +6.3%)	−1.5% (−9.0% to +7.0%)	1.000
MiniCog	60 (39%)	8 (20%)	**+18.7%** (+4.1% to +33.3%)	**+93.5%** (1.0% to +271.0%)	0.043
Pain management	153 (99%)	38 (95%)	+3.7% (−3.3% to +10.7%)	+3.9% (−3.0% to +12.0%)	0.187
Nerve block*	146 (94%)	26 (65%)	**+29.2%** (+14.0% to +44.4%)	**+44.9%** (+15.0% to +83.0%)	**0.000001**
Documentation of referral to orthopaedics	148 (95%)	37 (93%)	+3.0% (−5.8% to +11.8%)	+3.2% (−6.0% to +13.0%)	0.431

* Statistical significance *p* < 0.0026.

### 4.4 Comparison of Element Completion Between *EDs* When Pathway Was Used

Table 4 summarises differences in element completion between hospital EDs when the pathway was used. A comparative analysis was conducted to evaluate the implementation of key clinical interventions along the pathway across the three hospitals (**H1, H2, H3**). MiniCog assessment showed significant differences between **H1 versus H2** and **H1 versus H3** (*p* value < 0.00001). The *p*‐value of 0.0033 for the goals of care completion suggests that there is some difference in completion rates across the three hospitals. However, this value did not meet the significance threshold. All other interventions were consistent across hospitals, suggesting similar protocol adherence practices.

**TABLE 4:** Comparison of element completion between hospital EDs when the pathway is used.

Intervention	Hosp 1 (H1)	Hosp 2 (H2)	Hosp 3 (H3)	*p*‐value
Goals of care (GoC)	76/78	32/39	37/38	0.0033
Pain management	77/78	39/39	37/38	0.5926
Nerve block	75/78	36/39	35/38	0.5756
Documentation of referral to orthopaedics	75/78	35/39	38/38	0.0881
MiniCog*	57/78	2/39	1/38	< 0.00001

* Statistical significance *p* value < 0.0026.

### 4.5 Patient and Organisational Outcomes

Table 5 summarises the overall and individual hospital ED mean patient and organisational outcomes when a pathway was used and when it was not. None of the outcome differences reached statistical significance. This suggests that pathway use did not significantly impact waiting times, length of stay, or representation rates in this dataset.

**TABLE 5:** Overall, ED mean patient and organisational outcomes when the pathway was used and when it was not.

Outcome	Pathway Used	Pathway Not Used	*p*‐value
**Presentations**	**155**	**40**	
Door to Doctor (min)	60.0 (SD 58.7)	57.8 (SD 63.7)	0.8357
Door to X‐ray (min)	94.4 (SD 88.0)	106.0 (SD 138.5)	0.5150
Door to Blood (min)	151.3 (SD 137.8)	159.6 (SD 160.5)	0.7433
ED Length of Stay (hours)	9.9	9.7	0.6524
Hospital Length of Stay (days)	9.9	10.1	0.6524
28‐day Representation (%)	12 (8%)	4 (10%)	0.7460

### 4.6 Comparison of Patient and Organisational Outcomes Between EDs When Pathway Was Used

This section presents a comparative analysis of patient and organisational outcomes across three emergency departments (**H1, H2, H3**) where the clinical pathway was implemented. Statistical comparisons across the three hospitals revealed significant differences in several patient and organisational outcome metrics. Patients at **H2** (58.1 min) and H3 (58.3 min) were seen by doctors significantly faster than at **H1** (63.3 min), with *t* = 19.84, df = 58, *p* < 0.0001 and *t* = 19.00, df = 58, *p* < 0.0001 respectively. X‐ray wait times were longer at **H2** (106.4 min) and **H3** (103.1 min) compared to **H1** (93.2 min), with *t* = −52.17, df = 58, *p* < 0.0001 and *t* = −39.31, df = 58, *p* < 0.0001. Blood test wait times were shorter at **H2** (160.3 min) and **H3** (162.1 min) than **H1** (189.6 min), with *t* = 111.66 and 104.79, both *p* < 0.0001. ED length of stay was shortest at **H2** (8.5 h) and longest at **H3** (11.4 h), compared to **H1** (9.8 h), with *t* = 10.13 and −11.45, *p* < 0.0001. Hospital LOS was slightly longer at **H2** and **H3** than **H1**, with *t* = −4.78 and −5.58, *p* < 0.0001. No significant differences were found in 28‐day representation rates (**H1 vs. H2**: *p* = 0.7171; H1 vs. H3: *p* = 0.7268). These findings underscore **H2**'s performance and suggest caution in interpreting other observed differences. The results suggest significant differences in hospital performance and highlight potential areas for quality improvement (Table 6).

**TABLE 6:** Comparison of patient and organizational outcomes between hospital EDs when pathway is used.

	H1	H2	H3
Presentations that used pathway document (N)	78	39	38
Door to see Doctor (Min)*	63.3 min	58.1 min	58.3 min
Door to x‐rays (Min)*	93.2 min	106.4 min	103.1 min
Door to bloods (Min)*	189.6 min	160.3 min	162.1 min
ED LOS (hours)*	9.8 h	8.5 h	11.4 h
Hosp LOS (days)*	9.6 days	10.1 days	10.2 days
Representation N (%)	6 (8%)	2 (5%)	4 (11%)

* Statistical significance *p* value < 0.0026.

## 5 Discussion

This study retrospectively examined the use of the fractured NOF pathway, reporting on the use of the formal pathway document with secondary objectives of examining the completion of elements on the pathway, the impact of the pathway on patient and organisational outcomes, and differences between hospitals to inform local quality improvement initiatives. As part of a larger study focusing on identifying barriers to pathway use across Tasmanian EDs, our findings will inform post‐implementation improvement efforts at each hospital. Our results should be interpreted with caution, as it was unclear why there was an absence of evidence of completion and whether some missing element data were not completed or simply not documented. The absence of evidence of medication is rare and would, in our analysis, only include blank medication charts. Reasons for no analgesia in ED might include prehospital pain relief such that the patient is comfortable during their ED stay and/or the clinician thinking that a block was sufficient analgesia. The other reason would be that their pain needs were not addressed. However, results revealed high utilisation of the fractured NOF pathway in regional EDs, suggesting that current strategies for promoting use are largely successful. Our findings are likely to be generalisable to regional EDs and health systems similar to those in Australia. Opportunities to further understand these are warranted.

The importance of nerve blocks in limiting the use of opioids, and potentially decreasing delirium risk in elderly patients has been reported extensively [11]. Our study suggests that femoral nerve blocks were consistently used as pain management across the three hospitals demonstrating the clinical understanding and importance of completing this element within the pathway. Whilst it is important to highlight the possibility of systematic differences between patients in the pathway, which account for findings in this study, a study in Ireland reported similar findings [18]. Our retrospective study could not ascertain which profession or level of staff administered the femoral nerve blocks. There have been discussions as to which staff level is best suited to administer the nerve blocks. Traditionally, nerve blocks were administered by medical doctors, but recent evidence reveals that ED nurses with formal training can perform the procedure [19]. A systematic review suggested that nurse‐initiated protocols are effective in reducing time to pain management, investigations, and overall length of stay [20]. Other measures that have been suggested to reduce patient waiting include automatic notification of test results [21] which assist in speeding up diagnosis and treatment in the ED [22, 23]. Follow‐up studies may need to explore this area and perhaps provide clear delineation about individual professional roles and responsibilities within the pathway.

Patients who are not given pain management following fractured NOF are more likely to experience persistent pain after the injury, delirium, delayed mobilisation, and decreased health‐related quality of life [24]. Our study did not report on the 30‐min standard for pain management from first entry into the ED as per recommendations [25], and it was not clear from our retrospective data when a patient had pain management either prior or in the ED. However, the results provided information to complement local quality improvement initiatives. Follow‐up studies can focus on improving reporting as to whether patients are having timely pain assessment and treatment as per this quality‐of‐care standard.

Early identification of cognitive impairment in fractured NOF patients is crucial, as cognitive impairment is linked to poorer outcomes [26]. Reasons for low completion of cognitive assessments in our study are not clear, and the qualitative component of our follow‐up study will assist in understanding why. Between 32%–47% of patients with fractured neck of femur in Australian EDs, do not receive pain assessments. Cognitively impaired patients are especially affected, with only 45% assessed compared to 75% of cognitively intact patients [27]. Other studies have reported barriers to routine cognitive assessments in ED that include limited staff expertise, unclear responsibility for testing, time constraints due to high patient turnover, and competing priorities in patient care in this setting [28]. Validated screening tools help identify impairment early, allowing for tailored care and timely interventions. Routine screening can reduce complications, improve safety, and support better patient outcomes. Despite its benefits, cognitive screening is not yet standard practice in many EDs, highlighting a need for wider adoption [29]. Follow‐up studies may need to examine the contextual barriers to pathway use.

Our study revealed differences in pathway element completion between EDs despite the use of the same pathway document. Although these standards are uniform, local models of care can differ significantly [30, 31]. Perhaps staffing levels in busy periods need to be explored further in ED, as studies in Australia and the UK have reported longer waiting times for presentations outside standard operating hours [30, 31]. There is increasing pressure on EDs to meet national care standards. There is evidence of poor uptake of the Australia and New Zealand Guide and the Hip fracture clinical care standards [12]. Whilst prolonged ED stays are generally associated with higher mortality, it has been argued that the widely used four‐hour ED target lacks strong evidence, unlike time‐to‐surgery and early mobilisation benchmarks [5]. An observational UK study found that adherence to hip fracture care standards, including timely pain relief, imaging, and early geriatric input, was linked to improved outcomes [29]. A randomised controlled trial in Norway showed that direct admission to orthogeriatric units from EDs significantly improved activities of daily living up to 12 months post‐surgery [32]. Non‐randomised studies suggest benefits like reduced waiting times, shorter hospital stays, and fewer complications, though they often address only limited aspects of care [33]. Although time from presentation to operating theatre/surgical intervention was beyond this study's scope, it remains a critical outcome for future research. The Australian and New Zealand Hip Fracture Registry (ANZHFR) recommends surgery for neck of femur fractures within 48 h of hospital presentation, with early orthopaedic and anaesthetic assessment (within 4–8 h) and prompt pre‐operative optimisation, as delays are associated with poorer clinical outcomes [12].

The use of pathways potentially increases adherence to evidence‐based practices, which in turn improves the quality of care in the ED [34, 35, 36]. Implementation of pathways in EDs is complex and requires a multi‐faceted approach to understand the barriers and enablers in this context. As such, policy changes at state and local levels within the EDs are required to improve the use of pathways, assess and address issues of pathway compliance differences, and quality of care. Improving outcomes may require leadership, streamlined ED care, and better hospital‐wide flow and capacity [5]. The results of this study can be used to inform quality improvement initiatives at each ED.

### 5.1 Study Limitations

Retrospective studies cannot control for all potential confounding variables, such as staffing levels, severity of injury, or concurrent medical conditions, which could influence element completion, waiting times for investigations and treatment. The study relies on existing records, which may be incomplete or inaccurate, leading to potential biases or missing data. Whilst electronic health records (EHRs) hold great promise for improving clinical research and healthcare delivery, there is still much valuable information trapped in unstructured free text. To achieve meaningful use, systematic and automated methods are needed to extract actionable insights. Manual chart abstraction is prone to errors such as missing data, conflicting entries, vague or illegible notes, inconsistent coding, and transcription mistakes. These issues can compromise the accuracy of scientific investigations [37]. It is also possible that some patients may have ended up not having surgery or on a different pathway such as the palliative care pathway. The study's findings may have been limited by a small sample size and missing data. Key details like pain severity, assessment tools, and reassessment were poorly documented. Additionally, the timing between prescribing and administering analgesics was unclear, affecting the completeness of the results. This retrospective study could not accurately identify barriers to faster ED transfers due to limitations in health records. Potential delays may stem from late referrals from one ED to another, porter unavailability, staff workload, and unresolved medical issues requiring extended ED stays, all contributing to prolonged patient waiting times [5]. No information was available regarding the patients' English‐speaking abilities or co‐morbidities, such as dementia, which may have impacted completion of cognitive assessments. Further work and evidence are required to identify potential underlying causes of delays in investigations and increased ED length of stay. The Bonferroni correction reduces false positives but can be overly conservative, increasing false negatives and reducing statistical power. Chi‐square assumes large sample sizes and expected cell counts ≥ 5; it may be invalid with sparse data. Fisher's exact test is accurate for small samples but computationally intensive for larger tables.

### 5.2 Mitigation of Bias

We implemented comprehensive bias‐reduction strategies aligned with evidence [38]. Data were collected using a piloted, standardised instrument with predefined variables and a clear data dictionary. Three blinded, trained research assistants independently extracted data under academic supervision. Blinding to study hypotheses, regular calibration meetings, and consensus resolution of discrepancies ensured consistency. Chart reviews complemented digital records, and investigator roles were disclosed. Data were de‐identified prior to analysis. A representative sample was drawn from three diverse EDs using multiple patient identification methods. These measures enhanced transparency, minimised abstractor and selection bias, and strengthened methodological rigour.

## 6 Conclusion

This study suggests that pathway use was associated with significant increases in the administration of nerve blocks. Significant differences in pathway use, element completion, and associated waiting times were reported between hospitals. Findings support the pathway's role in standardizing care and guiding targeted quality improvement efforts.

## Author Contributions

The authors confirm contribution to the paper as follows: study conception and design: Innocent Tawanda Mudzingwa, Sarah Jane Prior, Phoebe Griffin, Emma Tavender, Viet Tran; data collection: Innocent Tawanda Mudzingwa, Sarah Jane Prior, Phoebe Griffin; analysis and interpretation of results: Innocent Tawanda Mudzingwa, Sarah Jane Prior, Phoebe Griffin, Emma Tavender, Viet Tran; draft manuscript preparation: Innocent Tawanda Mudzingwa, Sarah Jane Prior, Phoebe Griffin, Emma Tavender, Viet Tran. All authors reviewed the results and approved the final version of the manuscript.

## Ethics Statement

The study received Ethics approval from the University of Tasmania Human Research Ethics Committee (HREC)‐ Project ID: 28825 and site‐specific approvals from the three regions. A waiver of consent was sought and approved for use of medical record data. The results of this research will be published in a relevant journal and will be presented at relevant international scientific events in emergency medicine and implementation science.

## Conflicts of Interest

The authors declare no conflicts of interest.

## Data Availability

The data that support the findings of this study are available on request from the corresponding author. The data are not publicly available due to privacy or ethical restrictions.
